# Measurement of T Cell Alloreactivity Using Imaging Flow Cytometry

**DOI:** 10.3791/55283

**Published:** 2017-04-19

**Authors:** Stephen C. Juvet, Sajad Moshkelgosha, Sharon Sanderson, Joanna Hester, Kathryn J. Wood, Andrew Bushell

**Affiliations:** ^1^Division of Respirology, Departments of Medicine and Immunology, Toronto Lung Transplant Program, Multiorgan Transplant Program, Toronto General Research Institute, University of Toronto and University Health Network; ^2^Latner Thoracic Surgery Laboratories, Toronto General Research Institute, University Health Network; ^3^National Institutes of Health Research, Oxford Biomedical Research Centre, Translational Immunology Laboratory, NDORMS, Kennedy Institute of Rheumatology, University of Oxford; ^4^Transplantation Research Immunology Group, Nuffield Department of Surgical Sciences, John Radcliffe Hospital, University of Oxford

**Keywords:** Immunology, Issue 122, transplantation, immune synapse, imaging flow cytometry, tolerance, allograft rejection, T cell, antigen-presenting cell, dendritic cell

## Abstract

The measurement of immunological reactivity to donor antigens in transplant recipients is likely to be crucial for the successful reduction or withdrawal of immunosuppression. The mixed leukocyte reaction (MLR), limiting dilution assays, and *trans-vivo* delayed-type hypersensitivity (DTH) assay have all been applied to this question, but these methods have limited predictive ability and/or significant practical limitations that reduce their usefulness.**Imaging flow cytometry is a technique that combines the multiparametric quantitative powers of flow cytometry with the imaging capabilities of fluorescent microscopy. We recently made use of an imaging flow cytometry approach to define the proportion of recipient T cells capable of forming mature immune synapses with donor antigen-presenting cells (APCs). Using a well-characterized mouse heart transplant model, we have shown that the frequency of* in vitro* immune synapses among T-APC membrane contact events strongly predicted allograft outcome in rejection, tolerance, and a situation where transplant survival depends on induced regulatory T cells.**The frequency of T-APC contacts increased with T cells from mice during acute rejection and decreased with T cells from mice rendered unresponsive to alloantigen. The addition of regulatory T cells to the *in vitro* system reduced prolonged T-APC contacts. Critically, this effect was also seen with human polyclonally expanded, naturally occurring regulatory T cells, which are known to control the rejection of human tissues in humanized mouse models. Further development of this approach may allow for a deeper characterization of the alloreactive T-cell compartment in transplant recipients. In the future, further development and evaluation of this method using human cells may form the basis for assays used to select patients for immunosuppression minimization, and it can be used to measure the impact of tolerogenic therapies in the clinic.

**Figure:**
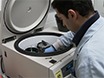


## Introduction

Solid organ transplantation has transformed the care of patients with end-stage diseases of the kidney, liver, heart, and lungs.**Owing to disparities in major and minor histocompatibility antigens, however, allografts are promptly rejected by recipient T cells if immunosuppressive drugs are not used. These agents have numerous adverse effects, including risks for cancer and organ dysfunction. A major clinical goal is therefore to lower the dose of immunosuppression to the minimum level required to prevent allograft rejection. This level is likely to vary depending on the degree of activation of the innate immune system; the degree of donor-recipient alloantigen mismatch; and inter-patient differences in immune function, pharmacokinetics, and pharmacodynamics.

Unfortunately, transplant clinicians do not have any tools for accurately assessing donor reactivity in individual patients^1^.**The mixed leukocyte reaction (MLR) can detect donor reactivity, but it fails to reliably predict graft outcome^2^,^3^. Limiting dilution assays, cytokine ELISPOTs, and the *trans-vivo* assay either measure a limited range of responses or are not practical^4^,^5^,^6^,^7^,^8^.Gene expression profiles have revealed signatures related to operational tolerance^9^,^10^,^11^,^12^ and rejection^13^,^14^,^15^, but these are not always generalizable across populations^16^ and may ultimately have limited usefulness in individual patients.**Sequence-based analyses of the T-cell receptor (TCR) of T cells in the peripheral blood^17^ or proliferating in the MLR^18^ have also been developed but require further validation.

Conceptually, it would be desirable to have an assay that detects the earliest requisite steps in recipient T-cell activation by a donor antigen.**Since culturing cells over days (as in the MLR) can introduce artefacts, such a test would ideally not require the measurement of downstream events, such as proliferation or effector function. Equally, however, it would also be desirable for the test to depend on some element of T-cell function, since purely descriptive assessments**(*e.g.,* TCR sequencing) may be unable to distinguish between anergic and functional T cells.

Numerous studies have indicated that prolonged T-APC contact is required for the formation of an immune synapse, which is an essential first step in the T-cell response^19^,^20^,^21^,^22^. We recently reported that, during dynamic *in vitro *time lapse imaging, about 5 - 10% of mouse CD4^+^ T cells form long-lasting contacts with allogeneic bone marrow-derived dendritic cells (BMDCs)^23^.**The frequency of prolonged contact was increased in animals that rejected a graft, whereas in mice previously rendered tolerant to the same antigens, it remained at levels seen in untransplanted mice^23^.**Prolonged interactions were reduced in the presence of recipient Tregs and increased in their absence, and we observed similar phenomena using human T cells and allogeneic monocyte-derived DCs (MoDCs)^23^.

However, the enumeration of prolonged contacts made within a polyclonal T-cell population is time-consuming and labor-intensive. We therefore made use of imaging flow cytometry to examine allogeneic immune synapse formation. Imaging flow cytometry incorporates the multiparametric data acquisition and analysis capabilities of conventional flow cytometry with the single-cell imaging abilities of fluorescence microscopy. This technique has been used by other investigators to study immune synapse formation by monoclonal T cells^24^,^26^,^27^or in the presence of superantigens^28^. In such settings, however, the frequency of responding T cells ranges from 30-100%, whereas alloreactive T cells are generally estimated to represent 5-15% of the total T-cell repertoire^29^,^30^,^31^,^32^. Importantly, we showed that imaging flow cytometry can produce a very comparable measure of alloreactive T-cell frequency^23^ and that changes in synapse frequency within a polyclonal T-cell population are predictive of graft outcome^23^. Currently, this approach has been optimized to measure the direct alloreactivity of CD4^+^ T cells, but, in principle, it could also be developed to examine CD8^+^ T cells and the indirect pathway. Indirect alloreactivity is believed to become increasingly relevant at longer times post-transplant^33^. We are currently developing this method to use human cells, which will allow for testing in patients. Thus, in the future, the overall approach may be useful for the functional evaluation of T-cell responses in transplant recipients before transplant; immediately after transplant; and in the long-term, when drug minimization becomes an important goal.

## Protocol

### 1. Prepare Reagents and Materials Required

1. Prepare phosphate-buffered saline (PBS) containing 2% fetal bovine serum (FBS) ("wash buffer"). Prepare PBS with 2% FCS containing 0.1% nonionic detergent ("perm-wash buffer"; see the Table of Materials). Prepare PBS with 1.5% formaldehyde. **NOTE: CAUTION!** Formaldehyde is corrosive and potentially carcinogenic and must be handled while wearing appropriate personal protective equipment.
2. Prepare PBS containing 2% FBS and 0.5 mM ethylenediaminetetraacetic acid (EDTA) for magnetic cell separation ("MCS buffer").
3. Prepare 50 µg/mL phalloidin-fluorescein isothiocyanate (Phalloidin-FITC) in dimethyl sulfoxide (DMSO). Prepare a 1 mg/mL nuclear stain (*e.g.* 1 mg/mL 7-aminoactinomycin D (7-AAD) in DMSO or bis-benzimide dye; see the Table of Materials) in DMSO. Prepare fluorochrome-labeled antibodies appropriate for the cells of interest and the imaging flow cytometer.
4. Obtain animal tissues (*e.g., *lymph nodes and spleen) as a source of T cells and allogeneic animal tissues (*e.g., *spleen and bone marrow) as a source of antigen-presenting cells or progenitors.
5. Prepare cell culture medium (*e.g.,* Roswell Park Memorial Institute medium (RPMI) 1640 or Dulbecco's Modified Eagle Medium (DMEM) supplemented with 10% FBS), 50 µM 2-mercaptoethanol, penicillin, and streptomycin and obtain 24- and/or 96-well cell culture plates.

### 2. Prepare Antigen-presenting Cells

**NOTE:** In theory, any APC population could be examined with this method. Immature mouse bone marrow-derived dendritic cells (DCs) as APCs were sued in this case. Many protocols exist for generating these cells (for example, References 34 and 35). Briefly, the following protocol was used.

1. Flush marrow from femurs and tibias into RPMI 1640 or DMEM.
2. Pass the suspended cells through a 70-µm cell strainer to remove small pieces of bone and debris.
3. Pellet the cells by centrifugation and then lyse red cells using an ammonium chloride buffer for 5 min at room temperature.
4. Pellet the cells by centrifugation (400 x g, 5 min) and resuspend the cell pellet.
5. Wash cells in 5 - 10 mL of wash buffer, pellet by centrifugation (400 x g, 5 min), and re-suspend the cell pellet.
6. Enrich hematopoietic precursors over a cell separation column by labeling the cells with biotinylated anti-CD3 (5 µg/mL), anti-B220 (5 µg/mL), anti-MHC class II (1 µg/mL), and anti-CD11b (5 µg/mL) antibodies.
7. Pellet the cells by centrifugation (400 x g, 5 min) and re-suspend the cell pellet.
8. Incubate the cells with anti-biotin magnetic microbeads (see the Table of Materials) at 4 °C for 10 min.
9. Wash and pellet the cells (400 x g, 5 min) and re-suspend them in 1 mL of MCS buffer before removing the labeled cells using a large positive selection (LS) magnetic cell separation column primed with 3 mL of MCS buffer and placed in its magnet. Wash the column 3 times with 3 mL of MCS buffer; the flow-through will contain the desired cells.
10. Culture the cells that pass through the column for 6 days in RPMI 1640 or DMEM supplemented with 2 ng/mL recombinant mouse granulocyte macrophage colony-stimulating factor (GM-CSF) and 2 ng/mL of recombinant human transforming growth factor β1 (TGFβ1). Replace half the medium every 2 days with fresh complete medium containing 2 ng/mL GM-CSF and TGFβ1. **NOTE:** Human TGFβ1 has activity in mouse cells. Data have been generated using these immature DCs. Other cells (*e.g.,* B cells and mature DCs) may be suitable as APCs but have not been tested in this assay.
11. Cryopreserve DCs in 90% serum/10% DMSO and store in liquid nitrogen; recover on the day of use. Prior to use, count the number of viable DCs in a hemocytometer using trypan blue exclusion. Re-suspend the cell pellet in culture medium at the appropriate density (see step 4.1) prior to use in section 4.

### 3. Prepare T Cells

1. Use negative selection methods to avoid inadvertently transmitting activating or inhibitory signals to the cells. **NOTE:** In this example, CD4^+^ T cells are prepared for analysis.
  1. To prepare CD4^+^ T cells from the mouse spleen, mash the spleen through a 70-µm cell strainer using the plunger of a syringe. Wash the cell strainer with wash buffer.
  2. Pellet the suspended cells by centrifugation (400 x g, 5 min) and then lyse the red cells by re-suspending the pellet in an ammonium chloride buffer for 5 min at room temperature.
  3. Pellet the cells by centrifugation (400 x g, 5 min) and re-suspend the pellet.
  4. Wash the cells in 5 - 10 mL of wash buffer and pellet by centrifugation (400 x g, 5 min). Re-suspend the pellet.
  5. Stain the cells with biotinylated antibodies to CD8, major histocompatibility complex class II (MHC II, 1 µg/mL), and CD19 (5 µg/mL). Incubate for 10 min at 4 °C.
  6. Wash the cells in 10 mL of wash buffer and pellet by centrifugation (400 x g, 5 min). Re-suspend the pellet.
  7. Incubate the cells with anti-biotin magnetic microbeads (see the Table of Materials) according to the manufacturer's directions.
  8. Wash the cells in 10 mL of wash buffer and pellet by centrifugation (400 x g, 5 min); re-suspend the pellet.
  9. Re-suspend the cells in 1 mL of MCS buffer and enrich the CD4^+^ T cells over a magnetic cell separation column primed with 3 mL of MCS buffer on a magnet. Wash the column 3 times with 3 mL of MCS buffer. Column flow-through will contain the enriched T cells.
2. By standard flow cytometry, assess T cell purity using an aliquot of the negatively selected cells. Stain the cells using a fluorochrome-streptavidin conjugate (to identify any biotin-labeled cells that should have been removed on the column) and an antibody or antibodies to identify the T-cell population of interest (CD4 in this case); a purity of ≥85% is acceptable^23^.
  1. Count the T cells in a hemocytometer by trypan blue exclusion (≥90% viability is acceptable). **NOTE:** MCS buffer contains EDTA, which must be removed prior to the assay. To accomplish this, pellet the cells by centrifugation (400 x g, 5 min) and wash in 1 mL of wash buffer. Pellet the cells again (400 x g, 5 min) and re-suspend in culture medium at the appropriate density (see step 4.1).

### 4. Co-incubate T Cells and DCs

1. Seed T cells and DCs at a 2:1 T:DC ratio in a 24-well or 96-well cell culture plate. Ensure that the final culture volume is ≤500 µL for 24-well plates or ≤50 µL for 96-well plates. **NOTE:** Smaller volumes encourage cell-cell interactions and allow space for subsequent fixation buffer.
  1. Adjust precise cell numbers empirically, but as a general guide, use 1 x 10^6^ T cells and 0.5 x 10^6^ DCs per well (96-well plate). These should be considered the minimum numbers of cells, because using fewer cells makes enumeration of immune synapses difficult.
    1. To increase cell numbers, set up replicate wells and pool *after* step 5 (fixation). **NOTE:** When setting up replicate wells, it is advisable to seed DCs in all of the wells first and then seed T cells in all of the wells; this minimizes discrepancies in incubation time between wells.
2. Incubate the plate for 4 h at 37 °C in a 5% CO_2_ atmosphere.

### 5. Fix Cells in Plate

1. Add 3 times the culture volume of 1.5% formaldehyde in PBS to each well and incubate at room temperature for 30 min; tt is important to fix cells prior to removing them from the plate to minimize the disruption of cell-cell interactions.
2. Transfer cells in the plate into tubes for subsequent washing and staining. At this stage, set aside additional cells for single-stain controls. Apart from these controls, the entire culture should be stained with the antibody cocktail (see step 4.1.1).

### 6. Stain Cells

1. Stain cells in 100 µL of wash buffer containing a cocktail of the desired fluorochrome-conjugated antibodies for 30 min at room temperature, protected from light. **NOTE:** The cocktail includes T cell-specific and APC-specific antibodies. Fluorochromes should be chosen such that they can be distinguished using the configuration of the imaging flow cytometer. In the experiments shown here, blue fluorophore-conjugated CD11b (5 µg/mL, see the Table of Materials) and APC-conjugated CD90.2 (5 µg/mL) were used.
2. Wash the cells in 1 mL of wash buffer and centrifuge for 5 min at 400 x g.**Decant the supernatant. Re-suspend the cells in perm-wash buffer containing phalloidin FITC at 0.05-0.5 µg/mL and incubate for 30 min at room temperature protected from light. **NOTE:** Phalloidin FITC concentrations of about 0.1 µg/mL work well for mouse cells, but the appropriate concentration is expected to vary by supplier, cell type, and imaging flow cytometer.
3. Wash the cells in 1 mL of perm/wash buffer and centrifuge for 5 min at 400 x g. Decant the supernatant. Re-suspend the cells in perm/wash buffer containing nuclear dye at the appropriate concentration (*e.g., *approximately 25 µg/mL 7-AAD) and incubate for 30 min at room temperature protected from light.
4. Wash the cells in 1 mL of perm/wash buffer and centrifuge for 5 min at 400 x g. Decant the supernatant. Wash the cells once in wash buffer, pellet, and re-suspend in 50 - 100 µL of wash buffer, transferring the cells to small, capped microcentrifuge tubes.
5. Proceed to data acquisition immediately or store the cells at 4 °C protected from light for up to several days prior to acquisition on the imaging flow cytometer. **NOTE:** Cells have been successfully stored in this way for up to 7 days. Longer storage may be possible but has not been tested.

### 7. Acquire Data

1. Initialize and configure the imaging flow cytometer according to the manufacturer's instructions. Ensure stability of the flow core prior to collecting any data.
2. Reserve one channel for brightfield image acquisition. Acquire single-stain control data with the brightfield channel turned off.
  1. Click the "Load" button and insert a tube containing a fully stained sample (a sample that has been stained with all required fluorochromes) into the holder.
  2. In the "workspace" window, select and create a new scatterplot with the aspect ratio over the area and gate singlets where the aspect ratio is close to 1. Create a new scatterplot for each channel used (intensity of the channel in the horizontal axis).
  3. For each fluorochrome, check the positive population and, if required, adjust the laser voltage in the "Illumination" box.
  4. Unload the tube and load the first single-stained tube.
  5. In the "Acquisition Settings" box, type the sample name and set the number of events that should be collected; if it is a single stain (for compensation controls), 1,000 - 2,000 events are sufficient.
  6. In the "Channels" box, select the channels that each sample has been stained with. For single-stain controls, all channels should be selected with brightfield and side scatter off. Click the "Record" button under the "Acquisition" box; when the number of events reaches the specified threshold, acquisition will stop automatically.
  7. Click the "Return" button to unload the tube. Repeat steps 7.2.4 - 7.2.6 for each single-stain control. Depending on the cytometer and software, it may not be possible to set all of the gates shown in **Figure 1** during acquisition (more precise gating is performed during analysis; see section 8).
3. Acquire samples as for single-stained samples (in step 7.2), but in the "Channels" box, select all channels that are required, including the brightfield channel.
  1. Before recording data, check to confirm that the channel intensity is appropriate for identifying the desired cell populations. If not, adjust the laser settings and re-record single-stain controls using the new settings, as described in step 7.2.
  2. For each sample, acquire several tens of thousands of events. **NOTE: **Under most conditions, cell-cell contact events are a small minority of the total cell number (most are single cells). In general, it is desirable to have at least 100 events in the final membrane contact gate.

### 8. Analyze the Data

1. Analyze the data acquired on the imaging flow cytometer using analytical software available for free from the manufacturer's website (a user account must be created; see the Table of Materials).

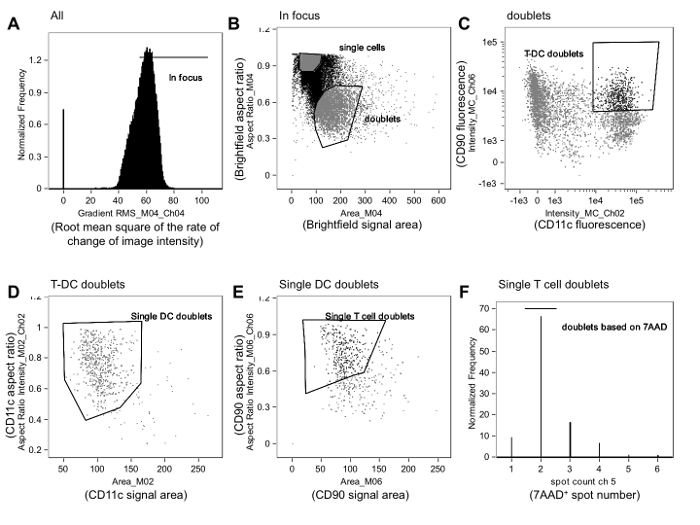
**Figure 1. Gating Strategy Used to Identify Alloreactive Immune Synapses. A. **In-focus events are gated from all events by reviewing cell images based on the root mean square of the rate of change of the image intensity profile (Gradient RMS) using the brightfield channel (Channel 4, Ch04), as described in the text. **B.** Among in-focus events, doublets are distinguished from single cells by plotting the aspect ratio versus area for the brightfield channel. Single cells are clustered close to the aspect ratio of 1 and have a smaller area, while doublets are close to 0.5 and have a larger area. **C*****. ***Fluorescence intensity of the APC (in this case, a dendritic cell [DC] marker, CD11c) is then plotted against fluorescence intensity of the T-cell marker (in this case, CD90.2), and double-positive events are gated. Borders of the gate can be refined by reviewing images of events near the borders. **D. **T-APC doublets are then refined so that they contain only one APC by plotting the aspect ratio versus the area of the APC marker (CD11c, Ch02). **E. **These single-APC doublets are then refined so that they contain only one T cell by plotting the aspect ratio versus the area of the T-cell marker (CD90.2, Ch06). **F.** Finally, events containing only two nuclei are selected by plotting a histogram of the spot count on the nuclear stain channel (7-AAD, Ch05) and gating events that contain only 2 7-AAD-positive spots (*i.e.,* nuclei). The events in this gate are analyzed for membrane contact and synapse formation, as described in **Figure 2**. The data were analyzed in a blinded fashion with respect to treatment assignment and are from a previously published experiment^23^. Please click here to view a larger version of this figure.

**NOTE:** The gating strategy is described in this section and is depicted in **Figure 1**. Analysis of imaging flow cytometry data should be performed in a blinded fashion with respect to treatment assignment. Although we believe that immune synapses and non-synaptic contacts are generally easily distinguished (see below and **Figures 2** and **3**), blinding should minimize bias arising from the subjectivity inherent to image analysis.

1. Generate a compensation matrix by loading the single-stain control data files into the compensation wizard. After loading the single-stained files, select the fluorescent channels used in the experiment. **NOTE:** The software automatically generates a compensation matrix, but this should be manually validated to ensure that the correct positive populations were selected.
  1. Double-click a value in the matrix and add the graph to the analysis area. Create a new gate if necessary to exclude any dead cells/doublets/false positives; this new refined positive population can be selected in the matrix box in the drop down menu for each channel.
  2. Repeat this for each channel.**Click on "Finish" to save the compensation matrix (.ctm file).
2. Obtain a data analysis file (.daf) from the raw file (.rif) by loading the .rif file into analysis software and applying the compensation matrix.
3. Once the data are loaded, perform gating to identify T cell-APC contact events. **NOTE:** A typical gating strategy involves identifying in-focus events, selecting doublets based on size criteria (the area versus aspect ratio of the event), and selecting T cell-APC doublets as events that are double-positive for the T-cell and APC markers (**Figure 1A-C**). **NOTE:** The aspect ratio is the ratio of the width of an event to its height, which allows discrimination of single cells (ratio close to 1) from doublets (ratio close to 0.5).
4. Identify in-focus events by plotting the root mean square of the rate of change of the imaging intensity profile (gradient RMS) in the brightfield channel (**Figure 1A**). Click on the gradient RMS histogram to display cell events in individual bins and then place a gate excluding out-of-focus events.
5. Plot the area versus the aspect ratio of the brightfield channel and draw a gate that identifies doublets based on event shape and size (**Figure 1B**). Review of images of events just inside and outside the border of this gate can help the analyst to refine the gate’s size and position. Then, construct a plot of these doublet events in which the intensity of the T cell marker appears on one axis and the intensity of the APC marker appears on the other.  Draw a T-APC doublet gate containing events positive for both markers.
6. Among the T cell-APC doublet events, select events that contain only one T cell and one APC.
  1. Do this by plotting the aspect ratio versus area for the APC marker and by gating on doublets containing a single APC (**Figure 1D**). Plot the aspect ratio versus area for the T-cell marker and gate on doublets containing a single T cell (**Figure 1E**). **NOTE:** Further refinement can be achieved by plotting a histogram of the spot count function as applied to nuclear stain fluorescence and gating on events with only two spots (**Figure 1F**).
7. In some cases, the two cells within a doublet will not be in contact; to identify cells in contact with one another, define object masks for APCs and T cells.
  1. Use the "Masks" option in the Analysis menu to open the Masks manager and define a new mask.**Type a name, such as "T-cell object mask." Click the "Function" button, and in the dialog box, choose "Object."
  2. Select the channel in which the T-cell marker is detected (*e.g.,* Ch06). Click "OK." **NOTE:** "Object (M06, Ch06, Tight)" is displayed in the Function box. This default object mask usually works well but may require optimization.
  3. Repeat this process to create an APC object mask in the appropriate channel.
8. After identifying the APC and T-cell masks, determine membrane contact by plotting T-cell marker fluorescence intensity in the APC object mask against APC fluorescence intensity in the T-cell object mask. On this plot, draw a gate that includes only cells in contact with one another. **NOTE:** Typically, there are fairly clear double-positive and double-negative populations (**Figure 2A**).**The border between the double-positive and double-negative populations can be set by reviewing brightfield images of cells in the border region to ensure that cells in contact are within the gate and that cells not in contact are excluded.
9. At this stage, manually review the phalloidin FITC images of events within this gate to distinguish mature immune synapses from simple cell-cell contact.**Use the "tag images" function to mark these images. **NOTE:** The percentage of tagged events (immune synapses) inside this gate can be used as an index of direct alloreactivity in the T-cell population being studied.

## Representative Results

This method was used to investigate CD4^+^ T-cell alloreactivity in mice rendered tolerant to donor alloantigens before heterotopic cardiac allograft transplantation.**CBA mice (H-2^k^) were given a tolerizing protocol consisting of a donor-specific (B6, H-2^b^) blood transfusion combined with a non-depleting CD4 antibody one month prior to receiving a B6 cardiac transplant. This protocol results in long-term allograft survival that is dependent on Foxp3^+^ regulatory T cells^36^,^37^. Seven days post-transplant, splenic CD4^+^ T cells were obtained from tolerized and non-tolerized recipients of B6 cardiac allografts and were co-incubated with B6 bone marrow-derived DCs according to this protocol. **Figure 2 **shows representative data from this experiment. The membrane contact gate is shown in **Figure 2A**, with green crosshairs placed on a synaptic event (left panel, 1) and on a non-synaptic event (right panel). **Figure 2B** shows the brightfield and fluorescence channels for this event. To reduce bias, data were analyzed by an observer blinded to treatment assignment^23^. As shown in several examples in **Figure 3**, both from non-tolerized****(**Figure 3A-B**) and tolerized (**Figure 3C-D**) CBA recipients of B6 hearts, synapses are easily distinguished from non-synaptic contacts by the presence of a dense FITC-positive ridge at the T-APC interface. These results show that visual detection of immune synapses made by recipient T cells tracks with the degree of alloreactivity in the recipient.


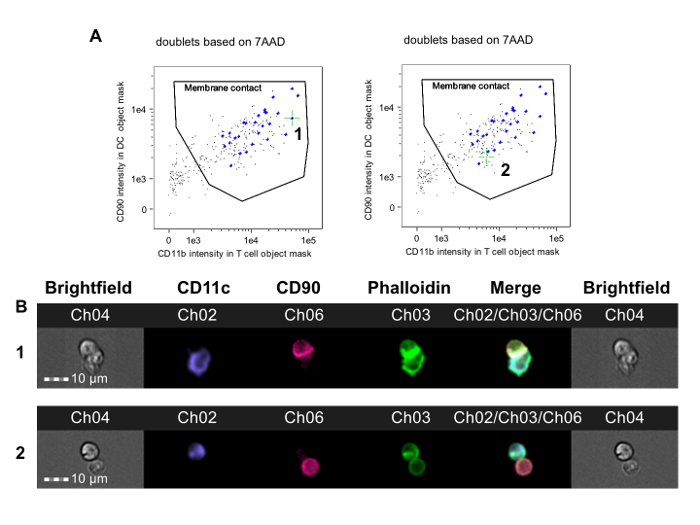
**Figure 2. Identification of T-APC Doublets with Membrane Contact and Immune Synapse Formation.** Events in the final doublet gate (**Figure 1F**) are analyzed. **A. **T-cell marker fluorescence in the APC object mask is plotted against APC marker fluorescence in the DC object mask.**Some doublet events have an APC and a T cell without cell-cell contact and appear in the lower left corner of the plot (images not shown). A membrane contact gate can thus be drawn that includes only doublets in which T cells and APCs are in contact. Images of each event in this gate are reviewed for evidence of actin cytoskeletal rearrangement in the phalloidin-FITC channel and can be tagged using the analysis software. The left panel indicates an immune synapse event (labeled 1 and indicated by green crosshairs), whereas the right panel indicates a membrane contact event without immune synapse formation (labeled 2 and indicated by green crosshairs). The determination of synapse formation requires manual review of these images, shown in B. **B.** The top row shows brightfield and fluorescence channel images for a doublet with an immune synapse (corresponds to event 1 in A); the bottom row shows a doublet with membrane contact but lacking synapse formation (corresponds to event 2 in A). The data were analyzed in a blinded fashion with respect to treatment assignment and are from a previously published experiment^23^. Please click here to view a larger version of this figure.


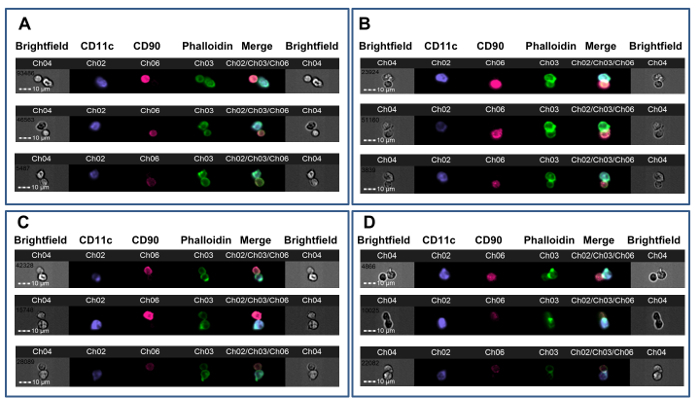
**Figure 3. Examples of T-APC Synapse Formation.** CBA mice received cardiac allografts from B6 donors after either no pre-treatment (**A-B**) or after tolerance induction with B6 whole blood under the cover of a non-depleting anti-CD4 antibody (**C-D**). After 7 days, splenic CD4^+^ T cells were tested for synapse formation with B6 DCs. **A. **Three examples of non-synaptic doublets with membrane contact from a non-tolerized animal. **B. **Three examples of immune synapses from a non-tolerized animal. **C. **Three examples of non-synaptic doublets with membrane contact from a tolerized animal. **D. **Three examples of immune synapses from a tolerized animal. Synapse formation is indicated by the presence of a bright, FITC-positive ridge at the T-APC interface (Ch03). The data were analyzed in a blinded fashion with respect to treatment assignment and are from a previously published experiment^23^. Please click here to view a larger version of this figure.

**Table:** 

**Antibody/Dye**	**Fluorochrome**	**Channel**	**Concentration**
CD11c	eF450	Ch02	5 µg/mL, titrate empirically
CD90.2	APC	Ch06	5 µg/mL, titrate empirically
Phalloidin	FITC	Ch03	0.05 - 0.5 µg/mL
7-AAD	-	Ch05	25 µg/mL

**Table 1. Antibodies and Dyes Used in this Study. **Fluorochrome-conjugated antibodies, dyes, suppliers, and recommended concentrations are presented in the table. The imaging flow cytometer channel that was used to detect each fluorochrome is also shown in the table.

## Discussion

Imaging flow cytometry has been used to demonstrate immune synapse formation between monoclonal T cells and APCs or in the presence of superantigens^24^,^25^,^26^,^27^,^28^. This method takes advantage of the fact that after a productive T cell-APC contact, the T cell rearranges its actin cytoskeleton, polarizing it toward the site of contact^21^. This rearrangement does not occur without TCR signaling, and it is therefore an early correlate of T-cell activation^19^,^20^,^21^. The method presented here adapts this approach to the measurement of alloreactive T-cell frequency in polyclonal T-cell populations. As such, it may in the future serve as the basis for the development of assays for donor reactivity in clinical transplantation.

Although direct comparisons have not yet been made, the detection of alloreactive immune synapses appears to have superior predictive power than the conventional MLR. For example, previous work has shown that, in the tolerizing protocol described above, the results of an MLR fail to reliably correlate with graft outcome^2^.

A number of assays have been developed for the operationally tolerant state in humans^9^,^10^,^11^, although these do not measure effector cell function in response to alloantigen.**In contrast, IFNγ ELISPOT assays^8^ measure effector T-cell function but cannot capture the full spectrum of cytokine secretion that may be relevant to acute and chronic allograft rejection, such as IL-17^38^,^39^.**The limiting dilution assay^4^, which is labor intensive, and the *trans-vivo* assay^6^, which requires mice, have significant practical limitations that would hinder their application in a clinical setting. Recent improvements on the analysis of proliferating cells using TCR sequence analysis of T cells responding in the MLR may be of value, but like the assay presented here, will require further validation in clinical studies^18^,^40^.

Further development of the immune synapse detection assay will require that a number of important questions be answered. First, the assay as developed only measures direct alloreactivity. The direct pathway involves the presentation of allogeneic MHC/peptide complexes on donor-derived APCs. The latter are generally eliminated quickly after transplantation, and further alloantigen presentation is carried out by recipient APCs presenting intact donor MHC (semi-direct pathway) or processed donor antigens on self MHC (indirect pathway). The indirect pathway is an important driver of chronic allograft rejection^33^,^41^.

In principle, it should be possible to detect indirect immune synapses using this assay, but indirectly alloreactive T cells have a much lower frequency than direct ones^42^,^43^, meaning that the analysis of a larger number of events will be required. A second consideration is that we have only tested this assay using CD4^+^ T cells, whereas CD8^+^ T cells are also an important component of the anti-donor response. Again, it should be possible to detect CD8^+^ T cell-APC synapses using this method. Another limitation is that the method requires the manual review and analysis of cell images in the final membrane contact gate, and we are currently working on the automation of this step.

Finally, the method requires testing and development in human subjects, and preliminary studies with human samples are currently being performed. Further phenotypic T-cell subset analysis (*i.e.*, effector, memory, regulatory, *etc.*) in combination with the detection of immune synapses in transplant recipients would represent a powerful approach for characterizing the alloreactive T-cell repertoire and will be an important focus for future work.

## Disclosures

The authors have nothing to disclose.
